# Impacts of warming on top-down and bottom-up controls of periphyton production

**DOI:** 10.1038/s41598-018-26348-x

**Published:** 2018-07-02

**Authors:** Garabet Kazanjian, Mandy Velthuis, Ralf Aben, Susanne Stephan, Edwin T. H. M. Peeters, Thijs Frenken, Jelle Touwen, Fei Xue, Sarian Kosten, Dedmer B. Van de Waal, Lisette N. de Senerpont Domis, Ellen van Donk, Sabine Hilt

**Affiliations:** 1 Leibniz-Institute of Freshwater Ecology and Inland Fisheries (IGB), Department of Ecosystem Research, Müggelseedamm 301, 12587 Berlin, Germany; 2 Netherlands Institute of Ecology (NIOO-KNAW), Department of Aquatic Ecology, Droevendaalsesteeg 10, 6708 PB Wageningen, The Netherlands; 3 Institute for Water and Wetland Research, Radboud University Nijmegen, Department of Aquatic Ecology and Environmental Biology, P.O. Box 90106500 GL Nijmegen, The Netherlands; 4 Institute of Freshwater Ecology and Inland Fisheries (IGB), Department of Experimental Limnology, Alte Fischerhütte 2, 16775 Stechlin, Germany; 5 Wageningen University, Department of Aquatic Ecology and Water Quality Management, P.O. Box 47, 6708 PB Wageningen, The Netherlands; 6 University of Utrecht, Department of Biology, P.O. Box 80.056, 3508 TB Utrecht, The Netherlands

## Abstract

Global warming profoundly impacts the functioning of aquatic ecosystems. Nonetheless, the effect of warming on primary producers is poorly understood, especially periphyton production, which is affected both directly and indirectly by temperature-sensitive top-down and bottom-up controls. Here, we study the impact of warming on gross primary production in experimental ecosystems with near-realistic foodwebs during spring and early summer. We used indoor mesocosms following a temperate temperature regime (control) and a warmed (+4 °C) treatment to measure biomass and production of phytoplankton and periphyton. The mesocosms’ primary production was dominated by periphyton (>82%) during the studied period (April-June). Until May, periphyton production and biomass were significantly higher in the warm treatment (up to 98% greater biomass compared to the control) due to direct temperature effects on growth and indirect effects resulting from higher sediment phosphorus release. Subsequently, enhanced grazer abundances seem to have counteracted the positive temperature effect causing a decline in periphyton biomass and production in June. We thus show, within our studied period, seasonally distinct effects of warming on periphyton, which can significantly affect overall ecosystem primary production and functioning.

## Introduction

Average global temperatures have risen by 0.6 °C during the last century and are predicted to increase by an additional 3–5 °C over the next century^1^. Ecological responses to climate change have been reported across various natural systems^2^, including shallow lakes (e.g.^3,4^), the most abundant type of freshwaters worldwide^5^.

One of the major processes potentially altered by global warming is primary production. Warming can elevate primary productivity as the rate of most subcellular reactions increase exponentially with temperature following the Van’t Hoff-Arrhenius relationship, wherein the calculated activation energy quantifies the change in reaction rate with temperature (Boltzmann 1872, Arrhenius 1889, as described in Allen *et al*.^6^). Increases in both biodiversity and biomass of planktonic algae in direct response to warming have been reported^7^. In turn, primary production affects key ecosystem functions of shallow lakes such as the regulation of greenhouse gas emissions^8,9^, carbon burial^10^, and consumer production^11^. Temperature-dependent physiological mechanisms also determine the nutrient stoichiometry of algae^12^, altering their quality as food for consumers^13^. Several studies have investigated the impacts of global warming on primary producers in shallow lakes either through a space-for-time approach^14–16^ or through temperature controlled mesocosm studies^17–22^. A major challenge of such investigations remains the determination of primary production by periphyton. This component can be most important in gross primary production (GPP) of shallow lakes, ponds, kettle holes, and also mesocosms due to these systems’ high surface area to volume ratios^11,23–25^.

While total primary production in aquatic ecosystems is usually determined by cost- and time-efficient diel O_2_ curves, the utility of this technique is limited in small and shallow ecosystems as it underestimates periphyton GPP^25,26^. Consequently, estimates of periphyton production at the whole lake scale have been hampered by low spatial and temporal resolution of productivity data^27^, while studies on the effects of warming on periphyton GPP are altogether lacking. Available studies on the impacts of temperature on periphyton biomass exhibit contradictory results. These have reported positive^14,16,20^, negative^28–30^, or non-significant effects^31^ of warming on periphyton biomass.

Warming may affect top-down effects through shifts in periphyton grazer community compositions, abundances, and activity rates^28,32,33^. Bottom-up effects may change during warming due to increased nutrient release from sediments^34,35^ and increased nitrogen loss by denitrification^36^, due to increased macrophyte surface for periphyton colonization^37^, and due to decreased light availability by enhanced phytoplankton growth^3^. These effects may differ in time leading to contrasting net effects of warming on periphyton biomass and production, yet studies with comprehensive within-system spatial and temporal resolution are lacking.

We investigated the impact of warming on shallow, freshwater ecosystem GPP and specifically periphyton with measurements every two weeks during spring and early summer (April-June). We applied a compartmental approach^24,25^ in 1000 L fishless indoor mesocosms with sediment, programmed to follow a temperate temperature regime (control) and a warm (+4 °C) treatment. In temperate shallow lakes, our studied period is decisive for the establishment of clear-water conditions stabilized by macrophytes which can be outcompeted by periphyton shading^38,39^. Furthermore, recent evidence shows that European lakes are warming up most significantly during spring^40^. We hypothesized that warming positively affects whole system and periphyton GPP during spring due to enhanced algal physiological rates. We expected warming to have an indirect positive bottom-up effect on periphyton GPP due to earlier nutrient recycling from fungal parasites facilitating advanced grazing of phytoplankton as shown in parallel studies^41,42^ and from higher mineralization rates in the sediment of the warm treatment. We also investigated, whether enhanced invertebrate grazing on periphyton in warmed treatments can reverse this trend leading to seasonally changing net effects of warming on GPP within the measurement period.

## Results

### GPP and biomass of primary producers

**Figure 1:**
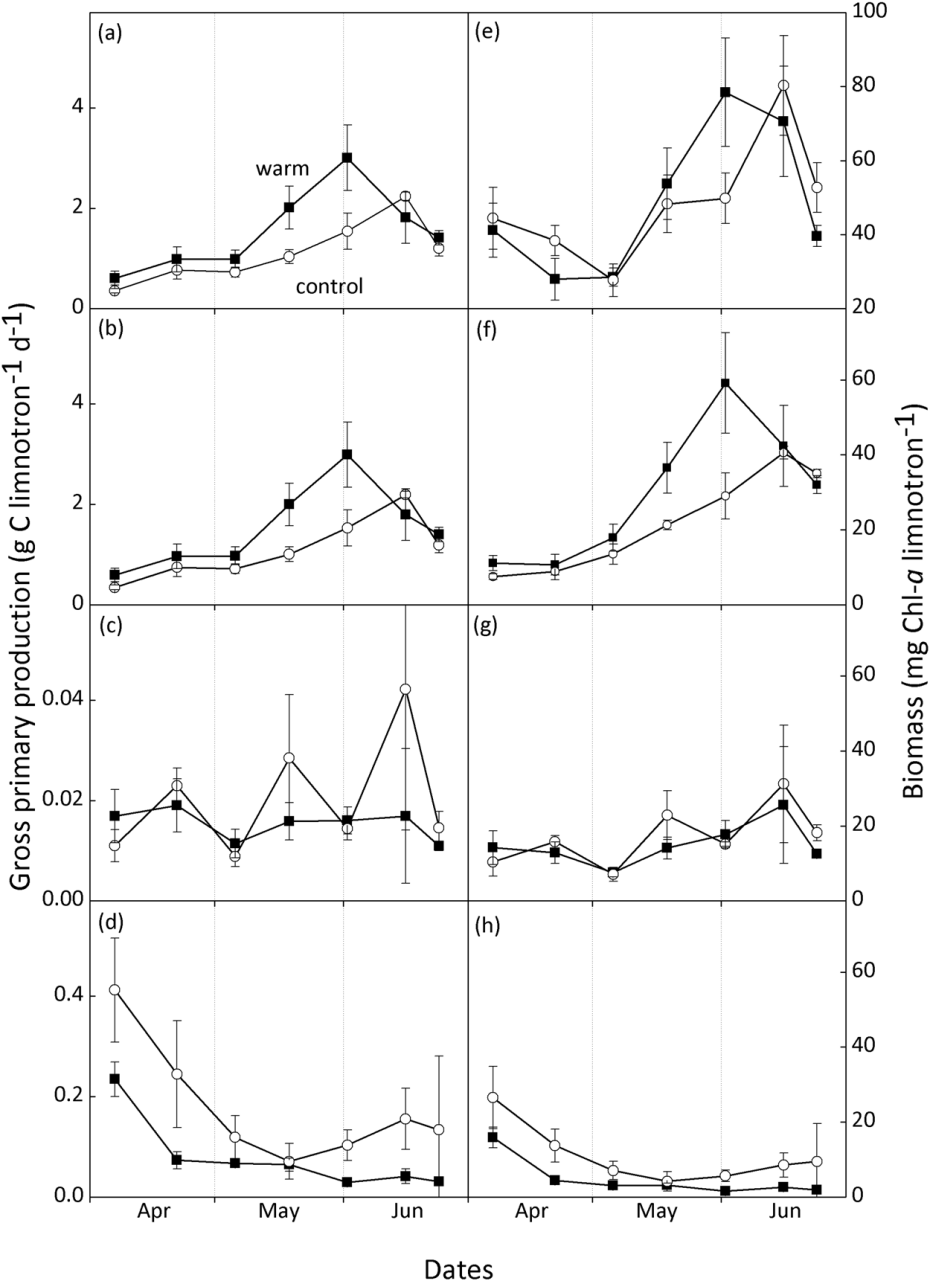
Gross primary production (GPP, left column) and biomass (chlorophyll-*a*, right column) of total primary producers (**a**,**e**), periphyton attached to the limnotron walls (**b**,**f**), epipelon (**c**,**g**), and phytoplankton (**d**,**h**) in control and warm (+4 °C) treatments. Values denote mean ± SE (*n* = 4).

On average, total limnotron GPP was not significantly different between the treatments during the investigated period (Fig. 1, Table 1). A maximum total GPP of 3.0 ± 0.7 g C limnotron^−1^ d^−1^ was recorded on 2-Jun in the warmed limnotrons, while the maximum was lower (2.4 ± 0.1 g C limnotron^−1^ d^−1^) and two weeks later in the controls (Fig. 1).

**Table 1:** Results of repeated measures ANOVA on the effects of treatment (+4 °C), time, and treatment x time interaction on periphyton biomass and GPP during the period of sampling (April till end of June).

Total PP		Biomass		GPP	
	*df*	*F*-value	*P*-value	*F*-value	*P*-value
Treatment	1	0.0	0.987	2.851	0.097
Time	7	22.42	1.38e-05***	36.572	1.38e-05***
Treatment x Time	7	0.003	0.956	0.209	0.649
Wall periphyton					
Treatment	1	4.488	0.0383* (+)	7.297	0.0095** (+)
Time	7	53.832	6.65E-10**	15.167	2.35E-10**
Treatment x Time	7	0.509	0.478	2.218	0.0492*
Epipelon					
Treatment	1	0.417	0.521	0.996	0.0323* (−)
Time	7	12.915	0.001**	2.003	0.0741
Treatment x Time	7	7	0.484	0.490	0.65
Phytoplankton					
Treatment	1	7.366	0.009** (-)	13.52	0.0006** (−)
Time	7	47.784	3.53E-09**	14.32	7.07E-10**
Treatment x Time	7	0.153	0.697	1.05	0.411

Plus (+) and minus (−) signs indicate the positive and negative effects of warming, respectively.

Overall, periphyton attached to the walls of the limnotrons (subsequently termed wall periphyton) contributed to 82–91% of the total limnotron GPP in the control and warm treatment, respectively during the investigated period (Fig. 1). The share of phytoplankton to overall limnotron GPP was low (17% and 8% in the control and warm treatment, respectively). Wall periphyton GPP increased until the beginning of June in both treatments. Similar to total GPP, the maximum of 3 ± 0.65 g C limnotron^−1^ d^−1^ was recorded on 2-Jun in the warm treatment and 2.2 ± 0.1 g C limnotron^−1^ d^−1^ two weeks later in the control (Fig. 1). Subsequently, wall periphyton GPP decreased in both treatments. Throughout the sampling period, wall periphyton GPP varied 0.8–1.9 fold between the treatments and was significantly higher in the warmed limnotrons (mean = 1.5 ± 0.4 g C limnotron^−1^ d^−1^) compared to the control (1.1 ± 0.3 g C limnotron^−1^ d^−1^) (Table 1). This warming effect was time-dependent (Table 1). To compare temporal trends between the two treatments, we plotted Weibull curves which showed that the warm treatment had a significantly earlier inflection point of increase as compared to the control (Table S1). Epipelon GPP, quantified by strips that rested on the sediment, was much lower than GPP produced by wall periphyton and showed no distinct temporal dynamics (Fig. 1). Epipelon GPP (about 1% of total GPP) was significantly lower in the warm treatment compared to the control (Table 1), averaging 0.015 ± 0.005 g C limnotron^−1^ d^−1^ and 0.020 ± 0.008 g C limnotron^−1^ d^−1^, respectively. Phytoplankton GPP was highest in March in both treatments and decreased henceforth, with an earlier decline in the warm treatment, which coincided with an advanced activity by fungal parasites^41,42^. Overall, phytoplankton GPP was significantly lower in the warm treatment (Table 1).

Total biomass of primary producers, expressed in chlorophyll-*a* (chl-*a*) was not significantly different between treatments (Table 1). Wall periphyton biomass exhibited similar patterns to its GPP rates described above (Fig. 1), and was significantly higher in the warm treatment, but was also time dependent (Table 1). Epipelon biomass showed less distinct dynamics, as its maximum values were lower than those of wall periphyton, and not different between the two treatments during the investigated period (Table 1). Phytoplankton biomass was significantly lower in the warm treatment between mid-March and end of June (Table 1).

### Effects of temperature on bottom-up control and stoichiometry of periphyton

**Figure 2:**
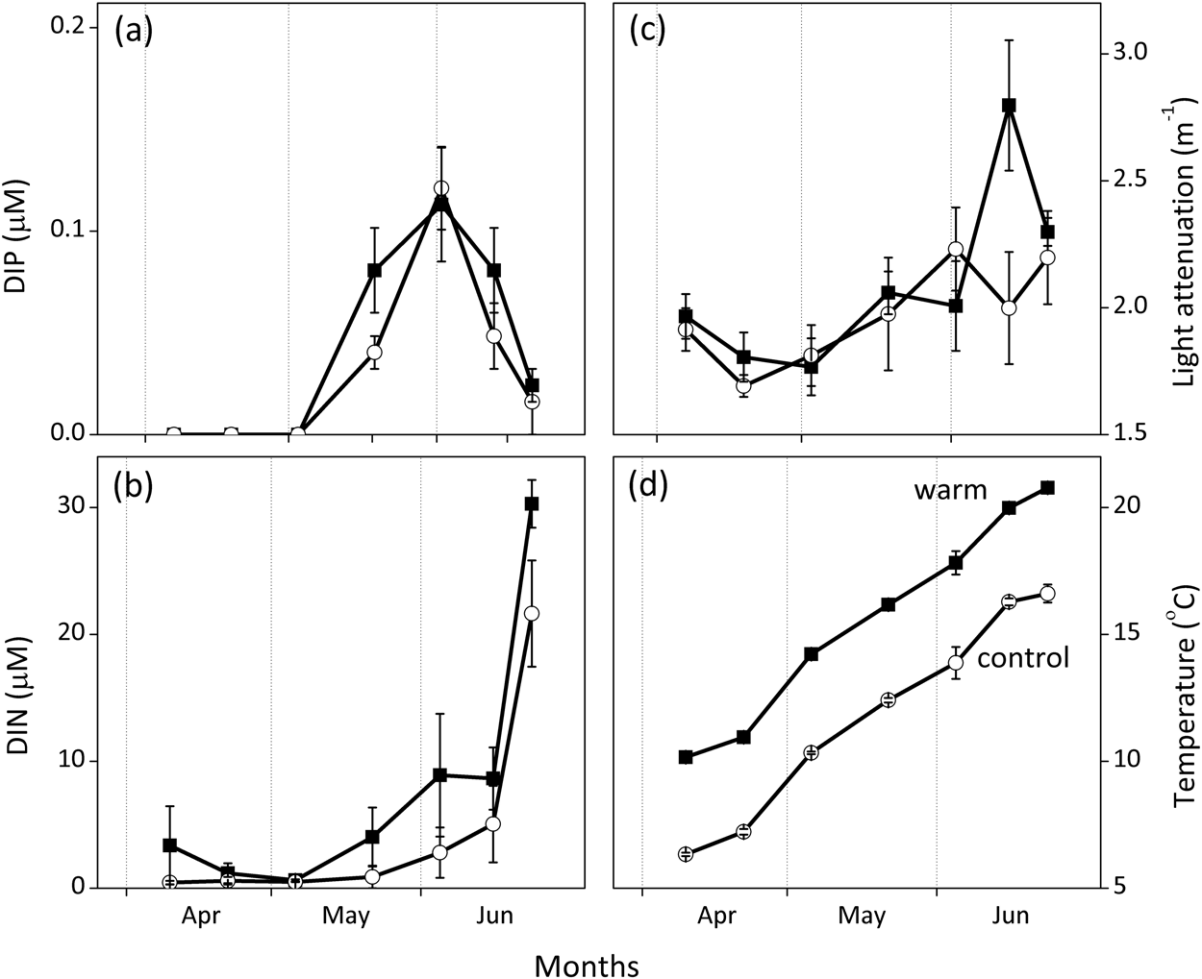
Concentrations of dissolved inorganic phosphorus and nitrogen (DIP, DIN), light attenuation, and water temperature in control and warm (+4 °C) treatments. Values denote mean ± SE (*n* = 4).

**Figure 3:**
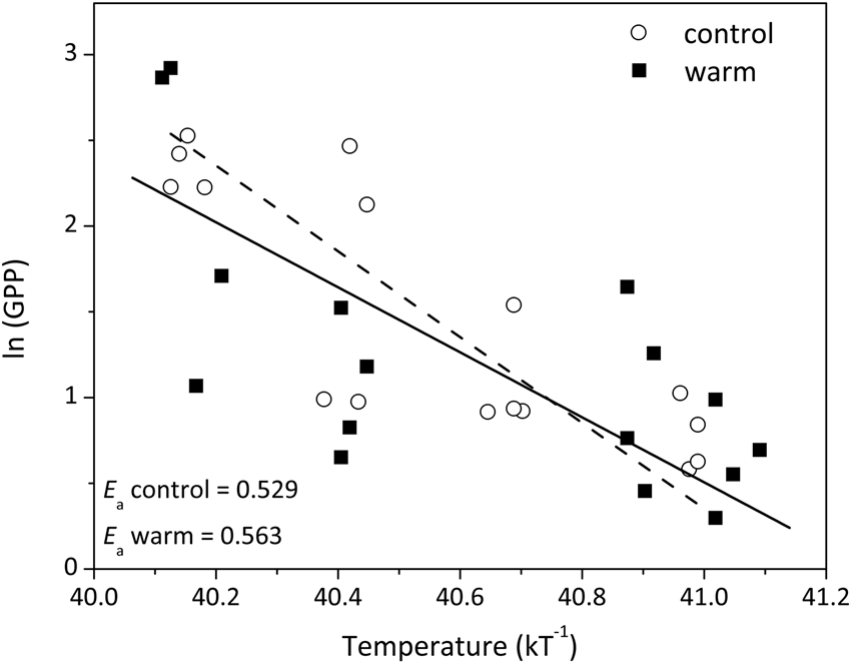
Arrhenius plots indicating temperature dependence of wall periphyton GPP between 7-Apr and 2-Jun, plotted as the relationship between log transformed GPP (originally measured in mg C m^−2^ d^−1^) and inverse temperature (kT^−1^), where k signifies the Boltzmann constant (8.61 10^−5^ eV K^−1^) and T denotes temperature in Kelvin.

During the investigated period, water temperatures rose from 5.8 to 17.5 °C in the control (average 10.8 °C) and 8.6 to 21.3 °C (average 14.5 °C) in the warm treatment (Fig. 2). To quantify the effect of temperature on gross photosynthetic rates we calculated the apparent activation energy (*E*_a_). Arrhenius plots focusing only on the initial period till early June, which depicted an increase in wall periphyton GPP, were similar for the control and warm treatment (Fig. 3). Specifically, the slopes of the regression lines fitted to this response were not significantly different (ANCOVA, *P* = 0.37, Fig. 3) and calculated activation energies (*E*_a_) were comparable, with 0.53 and 0.56 eV in the control and warm treatment, respectively.

Light attenuation (and thereby residual light availability) was not significantly different between temperature treatments (Table 2) and did not show drastic temporal fluctuations until June (Fig. 2). Dissolved inorganic phosphorus (DIP) concentrations were below detection limits in April and early May but increased in the second half of May and June (Fig. 2). Overall, DIP concentrations were not significantly different between treatments during the investigated period (Table 2). Dissolved inorganic nitrogen (DIN) concentrations were also not significantly different between the two treatments, though a marginal treatment and time interaction was detected (Table 2).

**Table 2:** Results of repeated measures ANOVA on the effects of treatment (+4 °C), time, and treatment x time interaction on dissolved inorganic phosphorus (DIP), dissolved inorganic nitrogen (DIN), and light attenuation during the period of sampling (April till end of June).

DIP			
	*df*	*F*-value	*P*-value
Treatment	1	0.111	0.74
Time	7	11.895	6.13e-16***
Treatment x Time	7	1.053	0.41
**DIN**			
Treatment	1	1.172	0.2818
Time	7	30.559	<2e-16***
Treatment x Time	7	1.839	0.0399*
**Light attenuation**			
Treatment	1	2.834	0.0988
Time	7	2.731	0.0181*
Treatment x Time	7	1.361	0.2436

**Figure 4:**
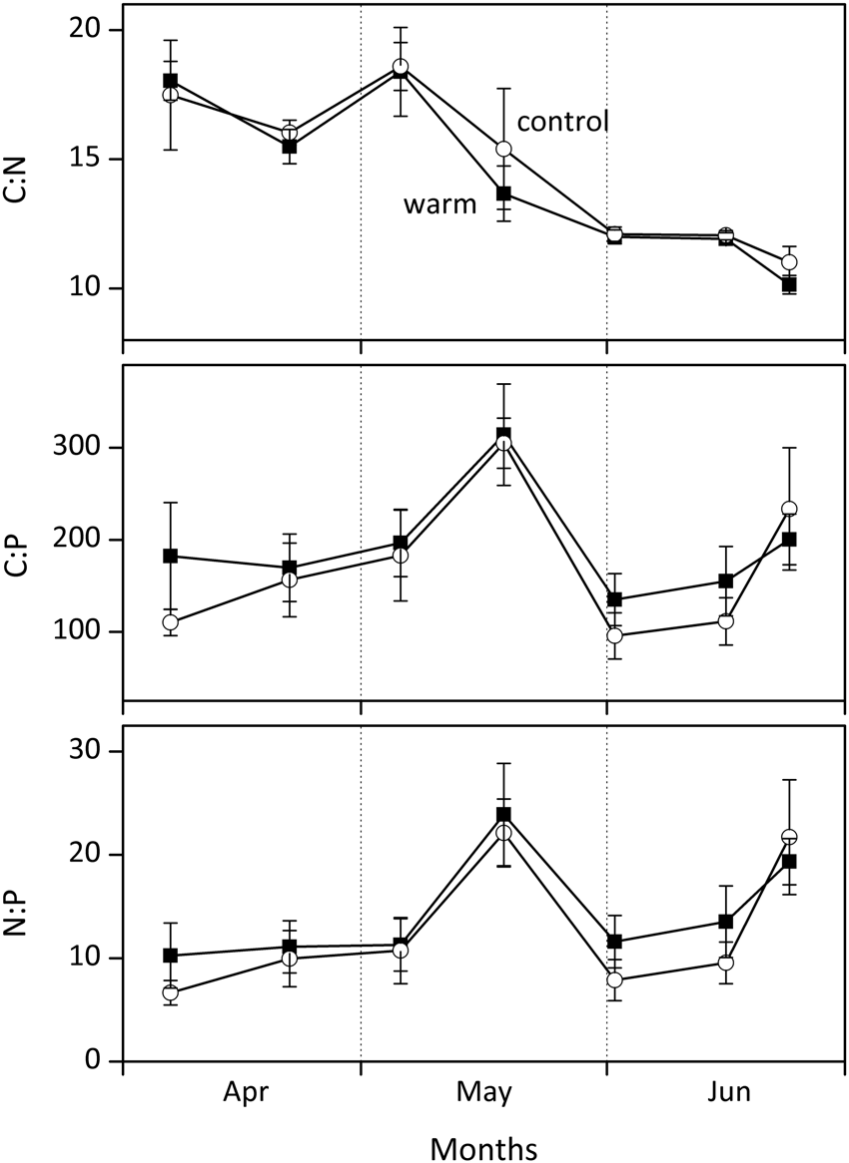
Periphyton elemental composition with C:N, C:P, and N:P molar ratios in control and warm (+4 °C) treatments. Values denote mean ± SE (*n* = 4).

**Figure 5:**
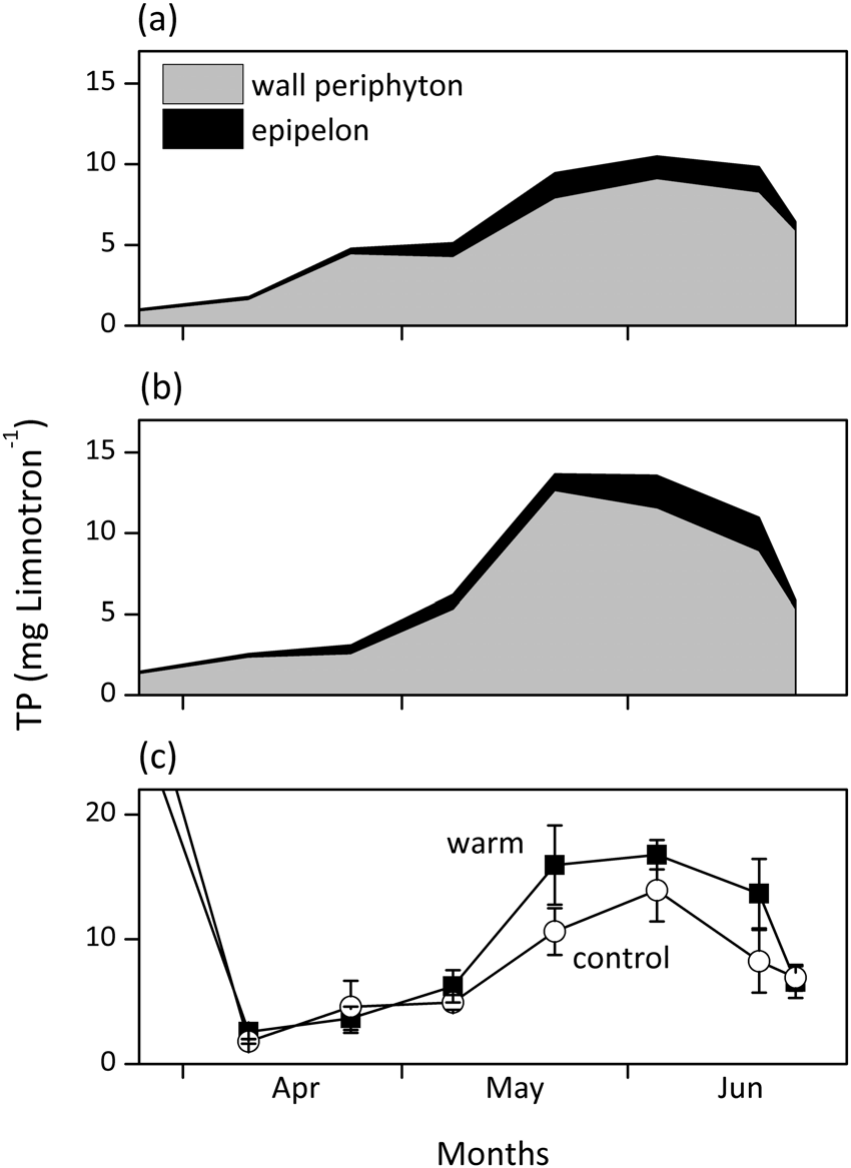
Total phosphorus (TP) stored in wall periphyton and epipelon in the (**a**) control and (**b**) warm (+4 °C) treatments. (**c**) pelagic-TP in the limnotrons of both treatments, calculated by summing up total dissolved inorganic phosphorus (DIP) in the water column and P content of all primary producers: wall periphyton, epipelon, and phytoplankton.

Periphyton elemental composition of carbon (C), nitrogen (N), and phosphorus (P) (C:N, C:P, N:P ratios) showed no significant differences between treatments during the investigated period (Fig. 4, paired Wilcoxon tests, *df* = 6, *P* > 0.05). C:N ratios showed a decline over time, but there were no clear trends in C:P and N:P ratios apart from a peak mid-May. Lower periphyton biomass buildup in the control treatment led to less P stored in wall periphyton and epipelon as compared to the warm treatment (Student’s *t*-test, *P* = 0.02; Fig. 5a,b). Total limnotron P stored in all primary producers also showed a higher peak in the warm treatment over the same period (Fig. 5c).

### Effects of temperature on top-down control of periphyton

**Figure 6:**
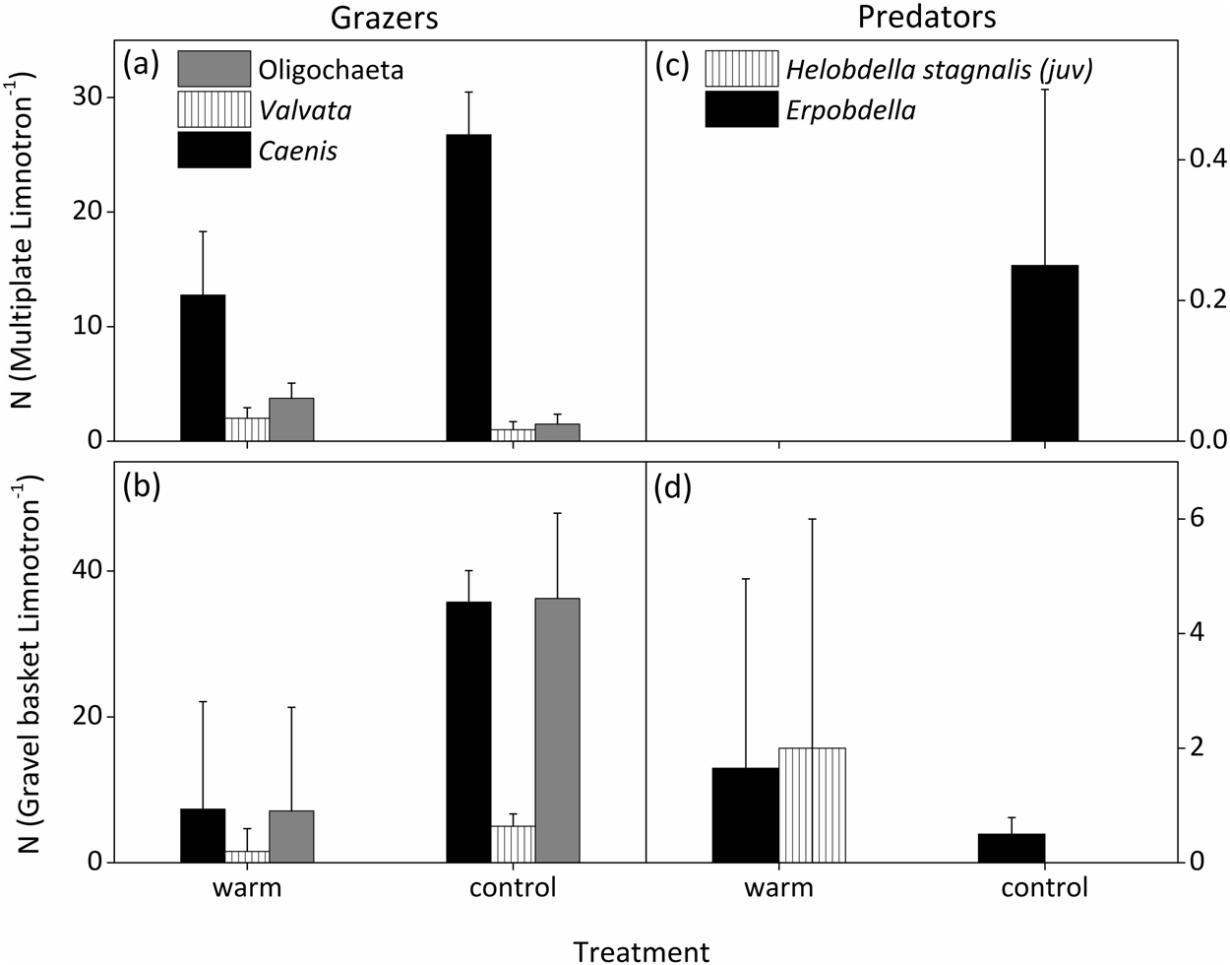
Most abundant herbivorous macroinvertebrates and their predators, sampled on 25-Jun from (**a**,**c**) multiplates and (**b**,**d**) gravel baskets in control and warm (+4 °C) treatments. Values denote mean ± SE (*n* = 4).

The periphyton-grazing macroinvertebrates, consisting of oligochaetes, snails, and mayflies (*Caenis*) and their predator leeches *(Erpobdella octoculata* and *Helobdella stagnalis*) (Fig. 6) were sampled in June. The most abundant snail genus was *Valvata*. In addition, single individuals of the species *Armiger crista* and the genus *Bithynia* were captured only in the warm treatment. The warm treatment had significantly lower abundance of *Caenis* (F_1,7_ = 6.416, *P* = 0.044 for gravel baskets, F_1,7_ = 4.837, *P* = 0.070 for multiplates) and abundances of *Valvata* tended to be slightly higher (F_1,7_ = 4.129, *P* = 0.088, *n.s*. for multiplates). Abundances of oligochaeta were not significantly different between treatments (F_1,7_ = 2.298, *P* = 0.18 for gravel baskets, F_1,7_ = 2.042, *P* = 0.203 for multiplates). The abundance of leeches (both *E. octoculata* and *H. stagnalis*) was higher in the warm treatment (Fig. 6).

## Discussion

In contrast to our hypothesis, average total GPP did not significantly increase in response to 4 °C warming in limnotrons where we simulated temperate lentic spring and early summer conditions, even though we recorded a higher peak production in the warm treatment. This was due to the contrasting effects of warming on phytoplankton and periphyton GPP and biomass during this period (Fig. 1, Table 1). Potential positive temperature effects on phytoplankton biomass were offset by an earlier termination of the spring bloom by fungal parasites facilitating zooplankton grazing^41,42^. In contrast, periphyton development was initially determined by bottom-up processes, while periphyton grazing seems to have significantly impacted its GPP only starting early summer.

**Figure 7:**
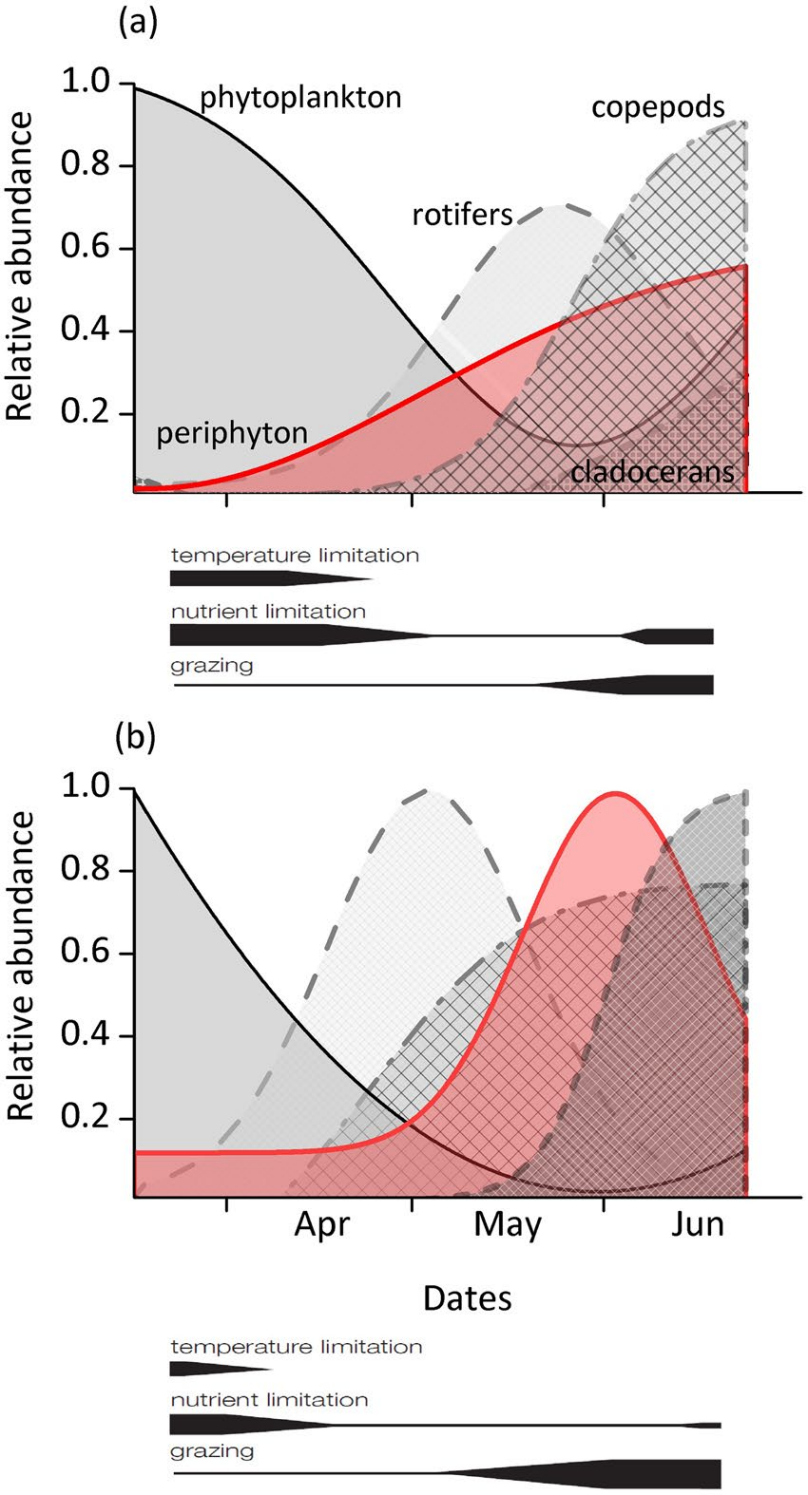
Dynamics of relative phytoplankton and periphyton GPP and the abundance of their potential zooplankton grazers (from^42^) in the (**a**) control and (**b**) warm (+4 °C) treatment. Data are retrieved from Weibull analyses and scaled to the maximum across treatment and within a group. Width of black bars below each figure indicates potential of limitation on periphyton GPP by each indicated factor. The period shown is extended into March to include the peak of the phytoplankton bloom which is described in more detail elsewhere^42^.

As expected, periphyton biomass and GPP in warm treatment were strongly enhanced compared to controls in spring (April and May) when water temperatures ranged from 6–16 °C. Higher spring temperatures nearly doubled maximum periphyton GPP, which was likely facilitated by higher P availability for periphyton in the warm treatments (Fig. 5c) originating from an earlier P release from phytoplankton grazing^42^ and a stronger P release from the sediment (Fig. S1). After the initial increase, periphyton biomass and GPP declined more rapidly in the warm treatment in June. The decrease in biomass coincided with an increase in the abundance of zooplankton (Fig. 7, see^42^ for further details on zooplankton abundances). This, along with stronger macroinvertebrate grazing pressure indicated by higher snail abundances in the warm treatment, counteracted the positive temperature effects, hence confirming our second hypothesis. As a consequence, differences in periphyton biomass and GPP between treatments disappeared by mid-June.

Limnotron primary production was dominated by periphyton due to the systems’ high surface to volume ratio, high phytoplankton grazing pressure due to lack of top-down control of fish on zooplankton, and the low light availability at the sediment surface (<2.5 E m^−2^ d^−1^) restricting epipelon GPP^43,44^. Periphyton GPP measured in the limnotrons are comparable to rates measured with a similar approach in small, temperate eutrophic lakes during the same seasons (0.7–0.8 g C m^−2^ d^−1^)^24^ and in hypertrophic fishless kettle holes (0.3–1.1 g C m^−2^ d^−1^)^25^. The overall high periphyton contribution to total GPP supports recent studies on the importance of periphyton production in both smaller^23,24^ and larger lakes^27,45^.

The fluorescence-based method applied for GPP measurements seems particularly useful for periphyton as it avoids problems of the more common O_2_ or ^14^C techniques. These are assumed to underestimate GPP in periphyton as some of the O_2_ produced or ^14^C fixed in periphyton is respired before reaching the oxygen probe or the end of the incubation period for ^14^C measurements^46–48^. PAM measurements might also underestimate GPP as fluorescence from deeper portions of thick periphyton layers might not be fully captured; but otherwise it could be argued the method used overestimated GPP as 1) actual light perceived by periphyton on the walls might be lower than light measured by the central flat sensor and 2) due to the direct inflection of excitation light in the PAM fluorometer as opposed to *in-situ* light scatter.

Arrhenius’ plots for the period of enhanced periphyton growth (April-May) showed that periphyton GPP responded to temperature following a similar pattern in both treatments, indicating that differences in periphyton growth between the two treatments might simply reflect the two-week lag of temperature in the control treatment. However, calculated apparent activation energy (*E*_a_) for periphyton GPP were 1.65 (control) and 1.75 (warm treatment) times higher than values predicted by the Metabolic Theory of Ecology (MTE^6^) explaining the relationship between temperature and biomass production at the ecosystem level. This disproportional increase in GPP likely points at a co-alleviation of another limiting factor in both treatments, further enhancing the temperature-driven periphyton production. The most likely factor is a higher P availability indicated by 1) declining periphyton C:P and N:P ratios in mid-May despite an increase in periphyton biomass and 2) by a slight increase in DIP concentrations during the same period (Fig. 2). However, P availability was likely higher in the warm treatment, stemming from an earlier termination of a phytoplankton spring bloom^41,42^ and a higher sediment P release during the investigated period (4–6 and 3.2–4.5 mg P limnotron^−1^ for the warm and control treatment, respectively). Differences in oxygen availability at the sediment surface^49^ and/or temperature-dependent mineralization rates^34,35,50^ could explain this TP discrepancy.

It has been shown that macroinvertebrates and zooplankton can both exert strong grazing pressure on periphyton. In our study, the stronger decrease of periphyton biomass and GPP in the warm treatment in June might be attributed to a higher abundance in periphyton grazing snails (*Valvata*), a two week advanced temperature optimum for snails^51^, and an advanced increase in zooplankton abundance^42^ (Fig. 7). Oviposition of *V. piscinalis* has been shown to occur between May and June^52^ suggesting a strong increase of their grazing impact after this period. As periphyton stoichiometry, and thereby their putative nutritional value^53^, did not differ between treatments, periphyton quality is not assumed to have led to differences in grazing pressure (Fig. 4). Furthermore, algal group composition was found to be similarly dominated by diatoms (HPLC analyses showed high pigment concentrations of fucoxanthin, data not shown) in both treatments in the beginning of June, when grazing started significantly reducing periphyton biomass.

Zooplankton can feed on periphyton, especially when phytoplankton abundance is low^18,54–57^. Zooplankton data^42^ show an advanced increase in rotifer abundance in May, and copepods and cladocerans in June in the warm treatment, coinciding with the decline in periphyton biomass (Fig. 7). As a result, zooplankton grazing pressure, expressed by the ratio of zooplankton biovolume to total chl-*a* values (phytoplankton and periphyton), increased from April to June in both treatments. The maximum ratio, however, was higher in the warm treatment (9.1 *vs* 5.7).

Other studies support the notion that warming affects grazer-periphyton interactions. For instance, positive impacts of temperature on periphyton were dampened - or altogether absent - in the presence of snails^58^ and the impact of grazing was stronger than nutrient availability^56^. Shurin *et al*.^28^ showed that the presence of planktivorous fish had a positive effect on periphyton, indicating that the decline in periphyton biomass in warmer temperatures was due to increased grazing activity. Similarly, Elster *et al*.^59^ reported decreased periphyton biomass with elevated temperature, likely due to increased consumption by chironomids. We thus conclude that the occurrence and the termination of an initially positive effect of warming on periphyton biomass and GPP in spring depend on type and phenology of periphyton grazers and their response to warming. The pattern observed in our experiment could be a likely scenario for temperate, fishless waterbodies (such as kettle holes and temporary ponds) and has important implications for their ecosystem functioning.

Our results suggest that in spring, warming may facilitate a stronger periphyton biomass build-up (Figs 1 and 7) which can hamper both phytoplankton through nutrient competition and macrophytes through shading^60–62^. In shallow lakes, losses of macrophytes induced by periphyton shading have been shown to result in regime shifts^38^ with potentially severe consequences for several important ecosystem processes, such as habitat provision, greenhouse gas emissions, C burial, nutrient retention and consumer production^63^. Depending on the type and phenology of the prevailing periphyton grazers, a facilitating effect of warming on periphyton grazers may not be sufficient to fully counterbalance the spring warming effects on periphyton, cascading to other ecosystem components. The temporal dynamics of warming effects on periphyton and their bottom-up and top-down control factors will thus be decisive for future ecosystem functioning of many temperate shallow water bodies. While climate induced changes in the phenology and subsequent mismatches in species interactions have often been studied in plankton communities^42,64^, benthic communities deserve more attention to arrive at a comprehensive assessment of global change effects in aquatic ecosystems.

## Methods

The experiment was performed in eight indoor limnotrons (mesocosms) of 1.37 m depth, 0.97 m diameter, as described elsewhere^42,65^. The limnotrons were filled in February 2014 with 908 L of tap water in addition to 80 L of pre-sieved sediment (5 mm mesh size to exclude large invertebrates) collected from a mesotrophic shallow pond (>90% volume) and an eutrophic pond (<10% volume) in Wageningen, The Netherlands. Each limnotron was spiked with a concentrated natural plankton assemblage (≥30 µm) retrieved from ~300 L water from the same pond as where the sediment was derived from. In addition, a small amount of plankton inoculum (<15% of spiked inoculum volume) and sediment (<1% of total sediment) was derived from another, more eutrophic pond (coordinates in DMS: 51°58056.7″N 5°43034.5″ E) to allow for a more diverse plankton community resembling different trophic states. Nutrients were added to each limnotron to ensure final concentrations of 86 ± 19, 2.4 ± 0.8 and 152 ± 37 (mean ± SD) µM of NO_3_^−^, PO_4_^3−^ and Si, respectively. Light of constant intensity (175 ± 25 μmol photons m^−2^ s^−1^) was provided by two HPS/MH lamps (CDM-TP Elite MW 315–400 W, AGRILIGHT B.V., Monster, The Netherlands) for each limnotron and followed the typical Dutch light: dark annual cycle.

The limnotrons were randomly divided into two groups of distinct temperature treatments (*n* = 4). The control treatment followed the average seasonal water temperature of Dutch lakes, while the warm treatment was 4 °C warmer in accordance with the IPCC RCP8.5 scenario that predicts a global temperature increase of 2.6 to 4.8 °C by the end of the 21^st^ century^1^. Water temperature was automatically recorded and controlled by a custom-made climate control system (SpecView 32/859, SpecView Ltd., Uckfield, UK). In addition, vertical profiles of each limnotron (temperature, light availability, turbidity and pH) were measured on a weekly basis (WTW Multi 350i, Geotech Environmental Equipment Inc., Colorado, US). Two oxygen loggers (HQ40d Portable probe, Hach, Colorado, United States) were circulated among the eight limnotrons to measure 24-hour oxygen diel curves. The initiation of the experiment was on 3-Mar (see Velthuis *et al*. (2017) and Frenken *et al*. (2016) for more details).

### Periphyton sampling

Periphyton was grown *in situ* on transparent polypropylene strips with textured surfaces (10 × 2.2 cm; IBICO, GBC, Chicago, IL, U.S.A.^62^) that were hung on plexiglass rods installed in the limnotrons on 16-Mar at three different depths below the water surface: 10 cm, 60 cm, and just above the sediment at 110–120 cm. This date marks the onset of our periphyton experiment when the polypropylene strips had no periphyton biomass (first timepoint, chl-*a* = 0). Four plastic strips from each depth were harvested first on 9-Apr and thereafter every two weeks until the end of June (*n*_harvest_ = 7). The collected strips were dark-adapted for 15 minutes prior to measuring rapid photosynthesis-light curves using a Phyto Pulse Amplitude Modulation (PAM) Emitter Detector Fiberoptics (EDF) unit (Heinz Walz GmbH, Effeltrich, Germany). Thereafter, we brushed periphyton off the strips using a toothbrush and pre-filtered limnotron water. The suspension was then filtered onto two 25 mm GF/F Whatman (Maidstone, U.K.) filters to determine chl-*a* concentrations and C, N, and P contents. We used chl-*a* values as a proxy for biomass of periphyton in the mesocosms. Filters were freeze-dried and stored at −80 °C until chl-*a* analysis by High Performance Liquid Chromatography (HPLC, Waters, Millford, MA, U.S.A.) following the procedure described in Shatwell *et al*.^66^. Periphyton hourly GPP was calculated following Brothers *et al*.^24^, using the equation:

1 $${{\rm{P}}}_{z}={{\rm{P}}}_{{\rm{\max }}}\cdot \mathrm{chl} \mbox{-} a(1-{{\rm{e}}}^{-{\rm{\alpha }}\cdot {\rm{Iz}}\cdot {\rm{Pmax}}-1})$$

where P_*z*_ is the production at depth z, P_max_ and α represent PAM-measured light-saturated photosynthesis and photosynthetic efficiency at low light, respectively, and I_*z*_ is photosynthetically active radiation at depth _Z_, calculated for every 10 cm depth following the Lambert-Beer equation:

2 $${{\rm{I}}}_{{\rm{z}}}={{\rm{I}}}_{0}\,\ast \,{{\rm{e}}}^{-{\rm{\varepsilon }}\ast {\rm{z}}}$$

where I_0_ is photosynthetically active radiation at the water surface and ε represents light attenuation (ε). Values from the rapid photosynthesis-light curves and chl-*a* of the strips deposited on the two higher depths (10 and 60 cm) were averaged to estimate wall GPP, whereas the lowest strips deposited on the sediment were used to estimate epipelon chl-*a* and GPP. Daily GPP was derived by multiplying calculated hourly GPP by number of light hours.

To determine elemental composition of the periphyton, filters were dried at 60 °C. A subsample of approximately 13% of the filtered surface area on the GF/F filter was folded in a tin cup (Elemental Microanalysis, Okehampton, UK) and analyzed for C and N on a FLASH 2000 NC elemental analyzer (Brechbueler Incorporated, Interscience B.V., Breda, The Netherlands). The remainder of the filter was combusted in a Pyrex glass tube at 550 °C for 30 minutes. Subsequently, 5 mL of persulfate (2.5%) was added and samples were autoclaved for 30 minutes at 121 °C. Digested P (as PO_4_^3−^) was measured on a QuAAtro39 Auto-Analyzer (SEAL Analytical Ltd., Southampton, U.K.).

### Phytoplankton sampling

Depth integrated water samples were taken using a tube sampler (1 m high; 3.5 L) and filtered over a 220 µm mesh to study phytoplankton biomass (as chl-*a*), community composition, and elemental composition. Chl-*a* concentrations were determined twice a week by fluorescence (Phyto-PAM, Heinz Walz GmbH, Effeltrich, Germany) but, in this particular study, only the data concurrent with periphyton sampling days were considered. Fluorescence measurements were calibrated by ethanol pigment extractions, followed by measurements with a photo-spectrometer (PerkinElmer, Groningen, The Netherlands). Linear regression of the ethanol extraction data and chlorophyll fluorescence (R^2^ = 0.60; *n* = 189) yielded a conversion factor of 0.87 to calculate chl-*a* concentrations from the fluorescence signal. Phytoplankton GPP was calculated following Brothers *et al*.^24^ for the dates corresponding to periphyton sampling, which were performed every two weeks. Full weekly phytoplankton biomass data and description is presented in^42^. We used a PAM Phyto-US unit (Heinz Walz GmbH, Effeltrich, Germany) to produce fluorescence-based rapid photosynthesis-light curves. P_z_ was calculated separately for each 10 cm layer using Eq. 1 with I_Z_ calculated for every 10 cm depth. Thereafter, total limnotron phytoplankton GPP was calculated by summing up P_z_ of all the separate layers.

### Sampling of macroinvertebrates

Multiplates and gravels baskets^67^ were used for collecting macroinvertebrates. On 13-May, the gravel baskets were placed on the sediment while the multiplates, each consisting of 10 layered hardboard plates (7.5 × 7.5 cm) with interspaces ranging from 0.5–1.5 cm, were hung halfway the water column against the walls of the limnotrons. On 25-Jun, each multiplate and gravel basket was carefully removed and extensively washed under running tap water over a sieve of 500 µm to remove the animals. All macroinvertebrates were identified alive to the highest possible taxon and counted. After identification, they were released in their respective limnotrons again.

### Sampling of inorganic nutrients

To determine dissolved inorganic phosphorus (DIP) and nitrogen (DIN), depth integrated water samples were taken twice a week and filtered over prewashed GF/F filters (Whatman, Maidstone, U.K.). Thereafter, concentrations of dissolved nutrients (PO_4_^3−^, NO_2_^−^, NO_3_^−^ and NH_4_^+^) were measured by a QuAAtro39 Auto-Analyzer **(**SEAL Analytical Ltd., Southampton, U.K.). When the concentration of nutrients measured was below the detection limit, we used a value equivalent to half the minimum detection concentration for each respective test. In this study, we show inorganic nutrient values of every two weeks on dates that are closest to periphyton sampling days.

To determine sediment P release, intact sediment cores (±6 cm) from all limnotrons were incubated in dark aquariums for one month, using temperature treatments of 6, 12, 22 and 30 °C. The cores were carefully supplemented with filtered limnotron water. The cores were subdivided to oxic and anoxic treatments (*n* = 3), which were purged with N_2_ gas until oxygen saturation dropped below 10%. After a settling period of one week, surface water samples were collected using Rhizon pore water samplers (Rhizon MOM, 0.15 µm pore size; Rhizosphere Research Products, Wageningen, The Netherlands) at five different times at day 0, 6, 10, 13 and 17 of the experiment. Water samples were analyzed for phosphate by an auto-analyzer (Skalar Sanplus Segmented Flow Analyzer, Skalar Analytical BV Breda, The Netherlands), and for TP by an ICP-OES (ICP-OES iCAP 6000 (Thermo Fisher Scientific, Waltham, USA).

### Statistical analyses

Treatment (+4 °C), time and their interaction effects on total and specific primary producer (wall periphyton, epipelon, and phytoplankton) biomass and GPP, in addition to DIP and DIN concentrations and light attenuation, were tested by repeated measures (RM) ANOVA, after checking for normality and homogeneity of variances in the samples and residuals (Shapiro-Wilk and Levene’s test, respectively). Throughout the study *n* = 8, with the first timepoint (16-Mar) signifying the start of periphyton colonization (chl-*a* = 0), followed by seven harvests. Uncertainties are reported as standard errors, unless stated otherwise. Additionally, to identify differences in seasonal timing, periphyton biomass and GPP calculated for each limnotron were analyzed with a Weibull function. In R 3.2.3^68^, we used the cardidates package^69^ fitweibull6 function, which fits a six-parameter Weibull function. These parameters are the offset before increase, inflection points of increase and decrease, slopes of increase and decrease and the maximum peaks. Each of these parameters was tested for significant differences between treatments using Welch tests. To check differences in elemental composition of periphyton between the two treatments, paired-sample Wilcoxon tests were used.

To test whether differences in periphyton GPP between the two treatments were proportional to temperature-related increase in subcellular reactions, the Van’t Hoff-Arrhenius relationship e^−E*a*/(kT)^ was fitted for the period of enhanced periphyton growth (April-May, 4 time points) and used to calculate the activation energy (*E*_a_, in eV) observed under different temperature conditions, where k is the Boltzmann constant (8.61 × 10^−5^ eV K^−1^) and T is the residing temperature of the limnotrons at any given time (in Kelvin)^6^. Under optimal growth temperatures, *E*_*a*_ of GPP for both cells and ecosystems is reported to be 0.32 eV^6^. The Arrhenius plots of the two treatments were fitted by least square regression lines, and their slopes were tested for significant differences using analysis of covariance (ANCOVA).

Periphyton nutrient stoichiometry differences between treatments were tested by Wilcoxon paired tests. The differences in abundance (log (abundance + 1)) of different groups of macroinvertebrates between both treatments were tested by univariate general linear models (GLM) using SPSS. All other statistics were performed using R version 3.2.3^68^.

### Data availability

The data analyzed during the current study are available from the corresponding author on reasonable request.

## Electronic supplementary material

Supplementary Information
