# Supplementary material for: Impacts of warming on top-down and bottom-up controls of periphyton production
Source: Sci Rep. 2018 Jul 2;8:9901. doi: 10.1038/s41598-018-26348-x (PMC6028635; doi:10.1038/s41598-018-26348-x)
Supplement: Supplementary file 1 — Supplementary Information [file 41598_2018_26348_MOESM1_ESM.docx]

**SUPPLEMENTARY INFORMATION**

**Impacts of warming on top-down and buttom-up controls of periphyton production**

Garabet Kazanjian*, Mandy Velthuis, Ralf Aben, Susanne Stephan, Edwin THM Peeters, Thijs Frenken, Jelle Touwen, Fei Xue, Sarian Kosten, Dedmer B. Van de Waal, Lisette N. de Senerpont Domis, Ellen van Donk, Sabine Hilt

****corresponding author***

|  | Value before increase | Point of increase | Slope of increase | Peak value | | Point of decrease | Slope of decrease | Area |
| --- | --- | --- | --- | --- | --- | --- | --- | --- |
| Biomass wall | 0.23 | 0.12 | 0.09 | | **0.03*** | 0.51 | 0.14 | 0.37 |
| GPP wall | 0.88 | **0.02*** | 0.17 | | 0.14 | 0.21 | 0.49 | 0.27 |

**Table S1**: *P*-values of the Welch tests comparing the Weibull6 fit parameters between the control and warm (+4°C) treatments. Values in bold indicate a significant difference between the treatments.

**Fig. S1**: Total phosphorus (in µmol m^-2^ d^-1^) released from the sediment in oxic and anoxic conditions.
